# The British Journals, &c.: Midwifery, and Diseases of Women and Children

**Published:** 1842-04

**Authors:** 


### MIDWIFERY AND DISEASES OF WOMEN AND CHILDREN.

Laceration of the Vagina and Escape of the Child into the Abdominal Cavity. In this case no instrument had been employed, nor had there been any violent manual interference, nor was the labour extraordinarily protracted or severe; yet rupture of the vagina took place, the womb remaining perfectly entire. The child passed into the abdomen of the mother, and was thence extracted by the Cæsarean section.—Mr. Blenkinsop, *Provincial Medical and Surgical Journal,* No. xi. *Dec.* 11, 1841.

---

Kiestein as a Sign of Pregnancy. The author detected it in 48 out of 50 cases, in the urine of pregnant women. In that of 17 non-pregnant women, he could not discover any trace of it.—Mr. Letheby, *Medical Gazette,* No. xiv. *Dec.* 24, 1841.

---

Fœtal Disease. The reporter saw occasion to perforate the head of a fœtus which could not pass, and he then extracted the child with the common forceps. Two quarts of sero-sanguinolent matter came away; and on examining the child's head, the remains of an enormous sac were discovered, which had widely separated the bones and compressed the brain, of which organ all that was found, was a quantity not exceeding two ounces. Notwithstanding this extensive disease of the cerebrum, and the operation, the child lived for fifteen minutes. From this case, the author infers the propriety of using the common forceps after perforation, as this operation does not appear to be necessarily fatal. In support of this view, he relates a case, in which, in consequence of an accident, he removed from a child of 7 years, a quantity of brain amounting to an inch square; yet recovery, without a single unfavorable symptom, followed.—Mr. Grantham, *Medical Gazette,* No. xiv. *Dec.* 24, 1841.

---

Knot on the Funis, causing Death of the Fœtus. A pregnant woman was seized with pains about three weeks before her time. By the use of proper means, parturition was postponed till the full time, when the child was born in a state of decomposition. The funis was soft and shrivelled, and there was a knot upon it so tight as completely to arrest the umbilical circulation. This accident doubtless took place in consequence of the uterine efforts three weeks previously. —Dr. Grieve, *London and Edinburgh Monthly Journal of Medical Science,* No. i. *Jan.* 1, 1842.

---

Presentation of the Upper Extremities, and Spontaneous Evolution (?) The author, after detailing a difficult case of his own, notices the great hazard both to mother and child, of a presentation of the upper extremities; yet happily this presentation is rare. Dewees had never seen a case of this sort. Collins could not find an instance of it out of 16,654 births in the Dublin lying-in hospital. Clarke, during his attendance in the same institution, found only one case reported (and this on the authority of a midwife), out of 17,922 births. As regards spontaneous evolution, Collins thinks this occurrence is so rare, that, in a practical point of view, it may be regarded as "almost fanciful." Denman, however, states that nature sometimes effects an evolution, and the author of this paper records one.

The following is a case in point, which confirms the experience of Denman and others, that in this presentation nature will sometimes, unaided, effect a delivery, and makes it conclusive that we are justified under some circumstances in considering the propriety of delay even in this most perilous condition, but I am satisfied we should never indulge such a hope except where the pelvis is very large and well expanded, or the child very small. On the 29th of April, 1838, I was called to Mrs. W———, the wife of an overseer on one of the plantations in my neighbourhood, who had been in labour during the preceding evening and night, under the management of an ignorant woman. On examination, I found the child's shoulder forced low, and firmly impacted in the pelvis; the hand and arm protruding, much swollen and livid ; pain almost constant; the uterus firmly contracted on its contents, so that the presenting part did not yield in the least to pressure; indeed, it was perfectly immoveable. The mother's pulse was feeble and very much hurried, but the uterine action continued most violent. I at once judged the only hope of relief to consist in lessening the body of the child by perforation. An experienced medical friend being near, I sought his assistance. He agreed with me exactly in regard to the deplorable condition of our patient, and expressed a doubt whether she could survive long enough for us to procure obstetrical instruments, which were not at hand. While we were expressing our views and sympathies, the midwife came from the adjoining room, and announced the safety of our patient. The child was delivered without aid, but whether evolution took place, or whether the child was forced out doubled up on itself I cannot say, as I was not in the room at the moment it was effected. The child was at the full period, large, and somewhat swollen. The capacity of the pelvis was very great. The women was of a healthy, vigorous constitution, and convalesced speedily. She had given birth to several children previously, and has also to one since, without the slightest difficulty.—Dr. TYLER, *Maryland Medical and Surgical Journal*, No. i. *July*, 1841.

---

CHINESE MIDWIFERY CASE. Some time ago, Pofa, the secondary wife of Mr. Chang, who lived at Hosham, a woman of tender years, though fully formed, when she became pregnant, labour came on at the eighth month; she had severe pains for several days, and the child was born, but shortly died; she again became pregnant, was prematurely delivered, and the child died as before. I then said that they must inform me when she was again in labour. The next year, at the eighth month, she was again in labour, which continued for three days, without success; when suddenly recollecting my words, they sent off post haste to call me; on the road I met the coachman, who said he was going to call her parents to take a final leave of her; when I arrived it was already night; on feeling her pulse I found it was not yet at the lowest ebb, and that she was still healthy. The midwife, on being questioned about the affair, said, that the child's head presented but could not come out. I ordered her to be put quickly to bed, and not to be disturbed, giving her at the same time a little medicine to soothe the womb. The next morning her husband came to me laughing, but did not speak. I asked the state of matters, and he said all is well. Yesterday, said I, the child's head was at the gate of life, how is it now? He answered, it has disappeared, and with a loud laugh went off. 120 days after this, that is at the term of twelve months after conception, she was delivered, and they said I was the child's father (ascribing the merit of saving its life to me), and it is now eight years old. And now I come to know, that on former occasions, they had pulled out the child by force, and it was only because the woman was young and strong that she survived.

In another part of this singular production, we are informed, on the authority of "the great pharmacopœia," "that pregnancy generally continues for seven or eight months, and sometimes for one or two years, and, in some rare cases, even for four years, and this should be made known!" Truly we may say the Chinese are a wonderful people!—*Chinese Treatise on Midwifery, translated by* Dr. LOCKHART. *Dublin Journal*, No. lx. *Jan.* 1842.

---

CROUP. The author, after detailing two fatal cases, observes: the important practical lesson, forced upon our notice by the cases just detailed, is the necessity of paying prompt attention to every case of hoarseness that occurs at this delicate age. To the superficial observer (as in both instances, I acknowledge, happened to myself) the affection may seem too slight to be made the subject of medical treatment, as it does not appear to interfere with the health or animation of the child, which continues to look as well, and play as merrily as ever, but not so in reality; the nature of the part attacked is such, and such is its structure, that before the disease has given sufficient warning of its approach its work is done, and medicine is unable to snatch the victim from destruction.

In speaking of the treatment adopted in the second case, I shall merely remark that I did not resort to general bleeding, both from the child's tender age (eight months), and because he had previously been weakened by leeches a few days before. Nor, indeed, am I disposed to think, that in cases where the disease is so purely local as it was here proved to be by the dissection, such a heroic remedy would prove generally eligible. Undoubtedly, in cases complicated with pneumonia, or even an extensive general bronchitis, it would be both desirable and necessary, but not at all so much so in a case only implicating the lining membrane of the larynx. My chief reliance, therefore, was in emetics and in mercury. In the selection of an emetic, I preferred hippo wine, as being equally effectual, and more safe than tartarized antimony, which I had found last winter to depress unduly the infants I had to deal with in the workhouse. Mercury, so much lauded in the treatment of this disease, I had recourse to, both because of its generally admitted power of checking or preventing the effusion of lymph in inflammation. and because I have experienced its great value in croup in other cases in which I have tried it. I gave it accordingly in large and repeated doses, and without any unpleasant effect, except that it did not accomplish the purpose for which it was exhibited. It was also employed as a dressing to the abraded surface of a blister, a method which I have found, on several occasions, highly satisfactory, in children of a very early age, threatened with hydrocephalus, and who were by this means immediately relieved from convulsions which had continued for two or three days from fever, and even from strabismus and coma.—Dr. Duncan, *Dublin Journal of Medical Science*, No. lx. *Jan.* 1842.

---

Ergot of Rye. In this paper the author endeavours to show, first, that ergot of rye, improperly administered, produces puerperal convulsions, "as a remote effect;" secondly, that it gives rise to the hour-glass contraction; and thirdly, that it gives a predisposition to hydrocephalus, in the early stage of infantile life. The chief or only cases in which the author appears to think it admissible, are, when there is serious hemorrhage from detachment of the placenta, accompanied with deficient uterine tone; and, in the latter stages of labour, in checking hemorrhage from whatever cause. Dr. Catlett, *Edinburgh Medical and Surgical Journal*, No. cl. *Jan.* 1, 1842.

---

Analysis of 265 Presentations in Labour.

*Table of the different Presentations in 265 Cases.*

Cranial, 260; breech, 3; feet, 1; placenta, 1. Total, 265.

*Cranial Presentations.*

| | | |
|---|---|---|
| Face presenting posteriorly ........ | —— | 197 |
| Face to right sacro-il. synch. .... | 195 | |
| Face to left sacro-il. synch. ...... | 2 | |
| Face presenting anteriorly ........ | — | 63 |
| Face to right acetab. ........ | — 1 | |
| Born with face to pubes .... | 1 | |
| Born with face to sacrum .. | 0 | |
| Face to left acetab. ............ | — 62 | |
| Born with face to pubes ...... | 4 | |
| Born with face to sacrum .... | 58 | |

Mr. Joseph Bell, *Medical Gazette*, No. xii. *Dec.* 10, 1841.

NEW SUBSTANCE, GRAVIDINE, AS A SIGN OF PREGNANCY. The fluid portion of the urine of pregnant women being drawn off, there appears a "natural sediment," which, whether held in solution, or separated by ether, has a striking resemblance to the serous globule; but when in a sedimentary state, bears an equally strong resemblance to the milk-globule in recent milk. This substance differs from albumen and caseum, the two animal substances most analogous to it: from the former, in being soluble in water by means of heat; from the latter, in being soluble by sulphuric and nitric acids. From gelatine it also differs: first, in being precipitated from its solution in water on cooling; secondly, though partially precipitated by tannin, the precipitate was soluble in water on boiling. The author calls it "gravidine," both from *gravidus* big with young, occurring as it does in pregnant women; and also from *gravis* heavy, seeing that it falls to the bottom of the vessel. Kiestein is but the pellicle which results from the decomposition of gravidine. As the globules forming the latter substance are decomposed, urates and purpurates are developed in the urine; and when these have broken up and assumed new combinations, the triple phosphates appear, with that beautiful crystalline appearance, described by Dr. Bird as one of the characteristics of kiestein.—Dr. STARK, *Edinburgh Medical and Surgical Journal*, No. cl. *Jan.* 1, 1842.

---

ON THE INCIPIENT STAGE OF CANCEROUS AFFECTIONS OF THE WOMB. Dr. Montgomery conceives that there is a stage of cancer of the womb which precedes that condition usually regarded as the first stage, but which is characterized by well-marked physical signs, and curable by appropriate treatment. The chief reason why practitioners in general have overlooked this fact is to be found in the neglect of vaginal examinations in cases where the earlier symptoms only of carcinoma are observed; and in this neglect they are confirmed by the common but erroneous supposition that the disease does not occur before a rather advanced period of life, and that its existence is incompatible with regularity in the menstrual function.

The general symptoms which indicate the incipient stage of carcinoma are sharp, but fugitive lancinating pains in the back and loins, across the pubes and shooting along the front of the thigh, or in the course of the sciatic nerve, producing numbness and sometimes debility of the whole limb. There often exists a fulness or decided tumour in one or other iliac hollow, with fixed pain and tenderness proceeding from the abdominal ring; the patient suffers from dysuria and has a sensation about the lower part of the rectum, as though she were affected with piles. Menstruation usually occurs regularly, but occasional bursts of hemorrhage take place in the intervals of the menstrual periods, and there is little or no leucorrheal or serous discharge. On an examination *per vaginam* the os uteri is found to project more than natural into the passage, it is irregular in form, and its margin is hard and often fissured. In the situation of the muciparous glands several small, hard projections may be felt, like grains of shot or gravel, under the mucous membrane, and pressure on these gives pain. With this there is usually a turgid state of the os uteri, which presents a deep crimson colour when examined with the speculum, while the projecting points have a blueish hue.

In this stage the disease often continues for years, the patient having only occasional attacks of pain and uneasiness with weeks of freedom from suffering. By degrees, however, the uterine symptoms become aggravated, the general health is impaired, and the signs of what is commonly called the first stage of cancer are found to exist. The vague complaints of the patient are often referred to rheumatism, sciatica, or gravel, and thus the time for affording relief is allowed to elapse unemployed.

Frequent examination has convinced Dr. Montgomery that the first discoverable morbid change, which is the forerunner of cancerous disease, takes place in and around the glandulæ Nabothi. The coats of the vesicles become thickened, and scirrhous matter is deposited around them, which gives to them at first the resemblance to grains of shot or gravel. The fulness in the iliac hollow is produced by the swelling of the ovaries or of the glands at the side of the uterus, a condition of much more frequent occurrence in all stages of carcinomatous disease than is usually supposed, and one the existence of which should immediately lead to the adoption of very decided measures

The treatment of this state of the uterus consists in the employment of local depletion, which will frequently need to be repeated, and in the administration of mercury so as slightly to affect the system. Iodine, the iodide of potassium or of iron may be afterwards used with advantage, and counter-irritation by blisters to various parts with the warm bath or hip bath will likewise be found of service. A degree of tenderness of the uterus sometimes remains behind after the graver symptoms have subsided. It is best relieved by the warm bath and by anodyne applications to the part, or a solution of the nitrate of silver. This can be applied by means of a bent glass tube of about an inch in diameter, which the patient, lying on her back, can introduce as far as its curvature, and then pour into the upper end the medicated solution, which will immediately flow to the os uteri and remain in contact with it until the tube is withdrawn, an advantage which is not obtained by any other method of applying lotions to this part.

Dr. Montgomery is convinced that the affection is curable by these means, while he has no doubt of its being an early stage of cancer of the womb. Of the truth of this fact evidence [?] was afforded on examination by Dr. Greene, of the body of a woman who died of carcinoma of the rectum. The fundus and body of the uterus were free from disease, but the lower part of the cervix and the os uteri presented precisely the characters above described, especially that of the feel as if they were grains of shot, or sharp gravel imbedded in its substance.

Dr. Montgomery, *Dublin Journ. of Med. Science*, Jan. 1842.

